# Comparison of Left Ventricular End-Diastolic Volume Approximated from Mean Blood Pressure and Stroke Volume and End-Diastolic Volume Calculated from Left Ventricular-Aortic Coupling

**DOI:** 10.3390/jcm13113204

**Published:** 2024-05-29

**Authors:** Takahiro Shiraishi, Yuka Matsuki, Yukiko Yamazaki, Kenji Shigemi

**Affiliations:** Department of Anesthesiology and Reanimatology, University of Fukui Hospital, Fukui 910-1193, Japan; tshira@u-fukui.ac.jp (T.S.);

**Keywords:** left ventricular end diastolic volume, Ees/Ea, preload, mean blood pressure, stroke volume

## Abstract

**Objectives:** The purpose of this study was to compare left ventricular end-diastolic volume (EDV), derived from left ventricular arterial coupling (Ees/Ea), and mean arterial blood pressure. Both of these methods of measuring EDV require some invasive procedure. However, the method of measuring EDV approximate is less invasive than the EDV coupling measuring method. This is because EDV approximate only requires arterial pressure waveform as an invasive procedure. **Methods:** This study included 14 patients with normal cardiac function who underwent general anesthesia. The point when blood pressure stabilized after the induction of anesthesia was taken as a baseline according to the study protocol. At the point when systolic arterial blood pressure fell 10% or more from the baseline blood pressure, 300 mL of colloid solution was administered over 15 min. EDV approximate and EDV coupling were calculated for each of the 14 patients at three points during the course of anesthetic. Each value was obtained by calculating a 5 min average. The timing of these three points was 5 min before, 5 min during, and 5 min after infusion loading. **Results:** The total number of comparable points was 42; 3 points were taken from each of the 14 participants. Both EDV approximate and EDV coupling increased through the infusion load testing. Scatter plots were prepared, and regression lines were calculated from the obtained values. A high correlation was shown between EDV approximate and EDV coupling (R^2^ = 0.96, *p* < 0.05). **Conclusions:** In patients with good cardiac function, EDV approximate can be substituted for EDV coupling, suggesting the possibility that EDV can be continuously and less invasively calculated under the situation of general anesthesia.

## 1. Introduction

**Figure 1:**
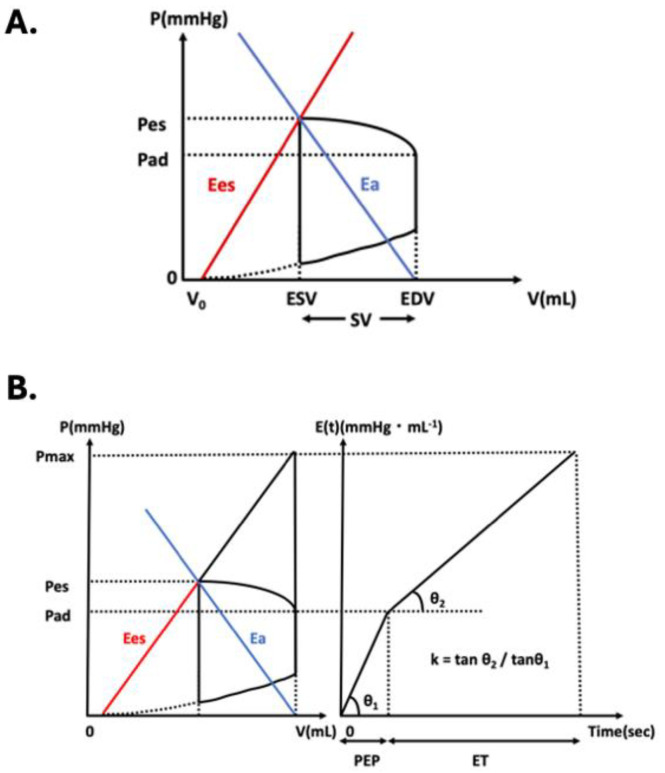
Left ventricular pressure-volume trajectory and left ventricular time-varying elastance. (**A**) The left ventricular pressure-volume trajectory is illustrated. Ees stands for left ventricular end systolic elastance and shows the left ventricular contractility, Ea for effective aortic elastance, and EDV for end diastolic volume. Ees/Ea is the barometer of left ventricular-arterial coupling and shows the balance between the left ventricle and the aorta that receives its output. These three parameters can be calculated using the equation below. Ea is Pes/SV, Ees is Pes/(ESV − V_0_). Pes: end-systolic pressure; Pad: diastolic pressure; Pmax: putative isovolumic pressure; SV: stroke volume; V_0_ is the volume when the left ventricular pressure is zero. (**B**) Two linear approximation lines of {E(t)} to calculate the constant (k) are shown. {E(t)} shows the time-varying elastance of a single cycle of the left ventricular contraction. Those two linear lines are the approximation of the pre-ejection phase and systolic phase. The ratio of the slopes of those two lines (tanθ_1_/tanθ_2_) is shown with a constant (k). The constant (k) is needed to obtain Ees/Ea using the method of Hayashi et al. PEP: pre-ejection period; ET: ejection time.

Increased preload is an important mechanism to increase effective circulating plasma volume. An increased effective circulating plasma volume leads to increased left ventricular filling pressure, thereby leading to increased cardiac output (CO) [1]. We view this mechanism as fluid responsiveness. Conventionally, many kinds of hemodynamic parameters are used to estimate the fluid responsiveness. Central venous pressure (CVP) is the one that describes the right ventricular preload, but it does not correlate well with CO or stroke volume (SV). Moreover, it necessitates the insertion of a central venous catheter and is highly invasive. Pulmonary artery wedge pressure (PAWP) is the most direct measure of left ventricular preload. Therefore, we can assess the fluid responsiveness more accurately than the other parameters; however, PAWP requires Swan–Ganz catheter insertion and is highly invasive, implying that it cannot be utilized easily in all patients as an exponent of fluid responsiveness. CVP and PAWP are the static indicators. However, SV variation (SVV) and pulse pressure variation (PPV) are dynamic parameters, which can be used as indicators of fluid responsiveness. They can be obtained easily because they do not require CVC or SG catheters, but they are less direct indicators compared to PAWP, and still need invasive arterial pressure monitor insertion. In addition, the patient must be under artificial respiration. The aforementioned indicators present advantages and disadvantages in monitoring fluid responsiveness. A major limitation is the invasiveness of monitoring; therefore, these parameters cannot be used for all patients undergoing general anesthesia. In view of this, we aimed to identify another indicator, which could show the fluid responsiveness and substitute CVP, PAWP, SVV, or PPV. It was preferable for it to be less invasive and applicable to all the patients. In addition, it needed to be a direct representative of the left ventricular preload to ensure an intuitive understanding of the parameter. Thus the development of a less invasive monitoring method was investigated for the purpose of easily monitoring left ventricular function for better hemodynamic management [2]. Therefore, we investigated Ees/Ea as a convenient barometer of the left ventricle. Ees/Ea represents ventricular–arterial coupling, illustrating the balance between the left ventricle and the aorta that receives its output [3]. This is expressed as the ratio of the end-systolic elastance (Ees) and the effective arterial elastance (Ea) in the left ventricular pressure–volume relationship (Figure 1A). Ees represents left ventricular contraction, while Ea represents left ventricular afterload. Ees/Ea reflects not only ventricular mechanical performance but also energetic performance [3]. As shown in Figure 1A, left ventricular end-diastolic volume (EDV) comprises the pressure-volume loop of the left ventricle and is a direct measure of cardiac preload [4], and EDV can be calculated when Ees/Ea is obtained. EDV corresponds to the sum of SV and end-systolic volume (ESV). The measurement of EDV classically requires the invasive procedure of placing a micromanometer in the left ventricle, which makes continuous, real-time monitoring difficult [5]. Ees/Ea, in contrast, can be estimated using the four following parameters: pre-ejection period (PEP), ejection time (ET), end-systolic pressure (Pes), and diastolic pressure (Pd) (Figure 1B) via the Hayashi method [3].

These four parameters can be obtained continuously using an electrocardiogram and phonocardiogram, arterial pulse waveform, and phonocardiogram [3]. We have previously reported that EDV measured from Ees/Ea correlates well with EDV measured from transthoracic echocardiography (TTE) [6]. In the present study, EDV was calculated from an approximation formula, using SV obtained with a FloTrac^TM^ (Edwards Lifesciences, Irvine, CA, USA) and mean blood pressure (Pm) (Figure 1A). Thereafter, EDV measured from Ees/Ea (EDV coupling) and EDV calculated with the approximation formula (EDV approximate) were compared, and their correlation was investigated. This study aimed to compare EDV coupling and EDV approximate.

## 2. Materials and Methods

**Figure 2:**
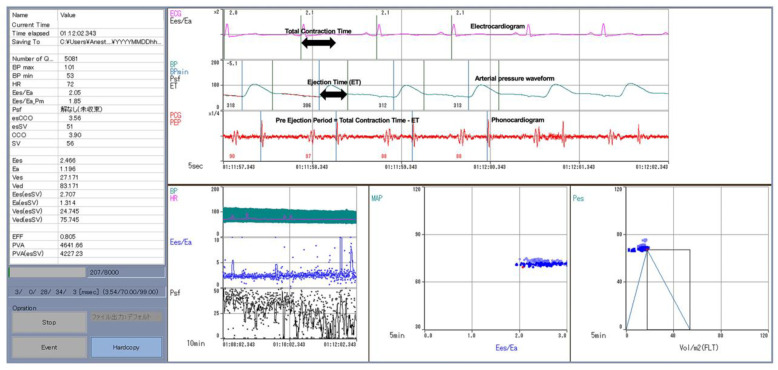
Measuring device screen.

This was a prospective, interventional study. The study protocol was approved by the University of Fukui Hospital research ethics committee (ethical code number-20220063, date of approval-2 August 2022) and was conducted in accordance with the Declaration of Helsinki. Written, informed consent was obtained from all the study participants. The participants were all adult patients with normal cardiac function (no angina, old myocardial infarction, congenital heart failure, or other heart diseases), during the period from July to October 2022, at the University of Fukui Hospital. This study was performed using electrocardiograms, radial artery pressure waveform, and phonocardiogram (Figure 2). After the induction of anesthesia, a radial artery cannula was inserted and connected to a FloTrac^TM^ (Edwards Lifesciences). This was primarily done to obtain SV and determine ET by measuring the time from the rise of the arterial pressure waveform to the dicrotic notch. This time interval showed the systolic phase of the left ventricle. A probe to measure esophageal temperature was also inserted orally. This was done for obtaining heart sounds. Heart sounds were obtained by amplifying sounds from a condenser microphone attached to the esophageal temperature measuring probe and transferred into a laptop for analysis. This probe enabled us to obtain the second heart sound (S2). S2 is heard when the aortic/pulmonary valve closes through auscultation. With this sound, we could calculate the PEP. We obtained the total contraction time by calculating the duration between the QRS wave of the electrocardiogram and S2. The difference between total contraction time and ET was the PEP. In addition to the PEP and ET, Pes and Pad were also needed to calculate Ees/Ea (Figure 1B). When left ventricular elastance E(t) is approximated linearly with the isovolumic contraction and ejection periods, the resultant slope ratio (velocity ratio of the decrease in left ventricular elastance) is defined as k. Diastole is unrelated, and the overall heart rate is not involved. The hypothetical left ventricular end-systolic pressure when the aorta is clamped, such that the ventricle does not eject into the arteries (peak isovolumic pressure; Pmax), is expressed as below using PEP, ET, and Pad.

Phonocardiograms are measured from an electrocardiogram, arterial pressure waveform, and a probe for measuring esophageal temperature that is inserted orally and connected to a condenser microphone. Pes, Pad, PEP, and ET are calculated from these measured data, and Ees/Ea is obtained.

Pmax = Pad + Pad(ET/PEP)·*k* = Pad{1 + (ET/PEP) · *k*} (1)

Meanwhile, as the increase in left ventricular pressure from Pes to Pmax and the increase in aortic pressure to Pes are attributable to the same ventricular ejection volume, the Ees/Ea ratio can be expressed as

Ees/Ea = (Pmax − Pes)/Pes(2)

From Equations (1) and (2), the theoretical equation

Ees/Ea = Pad/Pes·(1 + *k* · ET/PEP) (3)

is derived. Thus, if the value of *k* is known, the coupling value (Ees/Ea) can be calculated, as shown in Equation (4), using arterial pressure and systolic time measurements, without measuring left ventricular pressure–volume. If this theoretical equation and the experimentally obtained

*k* = 0.53 · (Ees/Ea)^0.51^ (4)

are simultaneously set up and solved using Newton’s method, Ees/Ea is obtained.

### 2.1. Method of Calculating EDV from Ees/Ea (EDV Coupling) [6]

EDV can be calculated using the definitions of Ees and Ea (Equations (5) and (6)), which can be understood using Figure 1A, to obtain their ratio (Equation (7)). This was rearranged as in Equation (8a) to calculate EDV.

Ees = Pes/(EDV − SV − V_0_) (5)

Ea = Pes/SV(6)

Ees/Ea = SV/(EDV − SV − V_0_) (7)

EDV = SV · (1 + Ea/Ees) + V_0_ (8a)

Because V_0_ is a much smaller volume than EDV, it can be treated as zero. Assuming that V_0_ is near zero in a healthy heart, EDV can be calculated using Equation (8b) [6].

EDV = SV · (1 + Ea/Ees)(8b)

The SV obtained from the FloTrac^TM^ is then substituted to obtain EDV. With these equations, we can obtain EDV with SV and Ees/Ea (Ea/Ees can be calculated as an inverse value of Ees/Ea.)

### 2.2. Method of Approximating EDV from Mean Blood Pressure and Stroke Volume (EDV Approximate)

The three elements that define systolic pressure (Pes) in the left ventricular pressure–volume relationship are EDV, Ees, and Ea (Figure 1A).

EDV = ESV + SV From Ees = Pes/(ESV − V_0_), (9)

ESV − V_0_ = Pes/Ees (10)

From Equations (9) and (10), Vo = 0, and with Pes as the mean blood pressure (Pm),

EDV = Pm · (Ees)^−1^ + SV (11)

Pes is the blood pressure at the point of the dicrotic notch, and mean blood pressure can be substituted to Pes according to Sunagawa [7].

When a reported value (2.3 mmHg·mL^−1^) [8,9] is used for Ees, Equation (11) is expressed as

EDV = SV + Pm/2.3.

**Figure 3:**
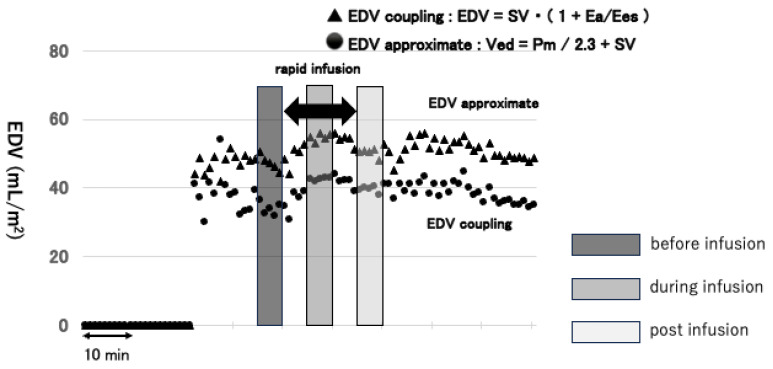
A representative case showing the time series of a fluid challenge. The figure shows the three time points at which data sampling was performed, surrounded by squares of different colors. In this case, with the administration of HES, both EDV coupling and EDV approximate increased. Although EDV coupling and EDV approximate decreased slightly, starting at the points during infusion and post-infusion, both the EDVs obtained at the post-infusion point were increased when compared with the EDVs obtained at the pre-infusion point.

The point at which blood pressure is stabilized after the induction of anesthesia was taken as the baseline. Hydroxyethyl starch (HES) 130/0.4 (Voluven^®^, mean molecular weight, 130 kDa; degree of molar substitution, 0.38–0.45; C2/C6 ration, 9:1, Fresenius Kabi Japan Inc., Tokyo, Japan) 300 mL was then administered over 15 min. HES was administered at the point when systolic arterial blood pressure fell 10% or more from the baseline blood pressure. Moreover, the mean values for EDV coupling and EDV approximate were obtained for each patient at three points (Figure 3). These three points were over 5 min immediately before the start of the infusion loading, 5 min in the middle of it, 15 min of infusion loading, and 5 min immediately after the end of the loading. In addition, the mean values of EDV coupling and EDV approximate were compared using a scatter plot and Bland–Altman plot. Patients who received vasoconstrictors at inappropriate times, i.e., from 15 min prior to infusion loading to 15 min after completion of loading, were excluded. EDVs in both cases were standardized for body surface area. Body surface area was calculated using the Du Bois formula [10].

### 2.3. Statistical Analysis

The statistical software package SPSS for Mac OS version 29.0.2.0 (IBM Inc., Chicago, IL, USA) was used. Continuous variables are described as means and standard deviations (SD), and categorical variables are reported as proportions. Simple regression analysis was used to compare EDV coupling and EDV approximate. Additionally, Bland–Altman analysis was used to assess the agreement between EDV coupling and EDV approximate. The bias (mean difference between EDV coupling and EDV approximate), the acceptable range of error, and the percentage error (PE) were calculated. The percentage error was calculated using the equation PE = 2SD of the bias/mean value using a method shown in a prior report. The agreement between EDV coupling and EDV approximate was interpreted to be clinically significant when the percentage error was ≤30% [11]. A *p*-value below 0.05 was considered statistically significant. As a sample size that would give a correlation coefficient of 0.5 with α 0.05 and power 0.8, and considering a drop-out rate of 20%, the number of points for analysis samples was set at 45, including drop-out cases with reference to a past report [6]. The patients enrolled numbered 14; however, 3 points of obtaining EDV coupling and EDV approximate were taken for each patient, thus expanding the sample size to 42.

## 3. Results

### 3.1. Patient Background

A total of 14 patients (7 men, 7 women) were included in the analysis, and the number of points for the analysis of participants was 42. The patients’ characteristics are shown in Table 1. The patients’ mean age was 63 ± 14 years, the height was 162 ± 8 cm, the weight was 62 ± 11 kg, PEP 86 ± 62 s, the ET was 330 ± 60 s, EDV coupling was 51 ± 11 mL/m^2^, the EDV approximate was 56 ± 7 mL/m^2^, and the SV was 64 ± 10 mL. The anesthesia method used was general intravenous anesthesia in six patients, intravenous anesthesia combined with epidural anesthesia in seven patients, and inhalation anesthesia in one patient. The physical status of all participants was considered to be class 1 or 2 according to the American Society of Anesthesiologists class. 

### 3.2. A Representative Example

Figure 3 shows one representative case of fluid infusion. With the administration of fluid, EDV coupling and EDV approximate increased. Although EDV coupling and EDV approximate slightly decreased during infusion and post-infusion, both the EDVs obtained at the post-infusion point were increased compared with the EDVs obtained at the pre-infusion point (Figure 3). 

### 3.3. Correlation Analysis

**Figure 4:**
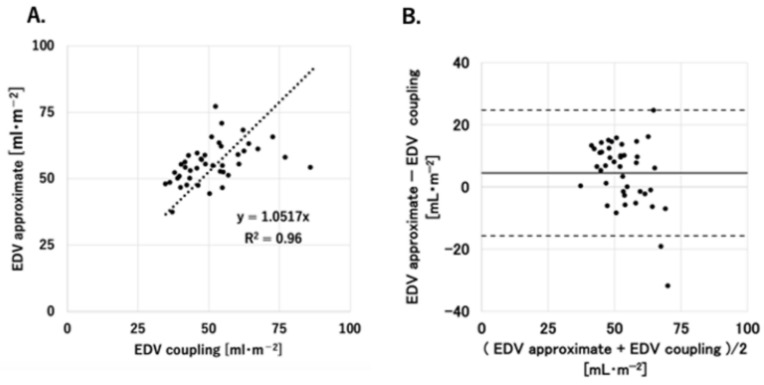
(**A**) Correlation diagram (horizontal axis−EDV coupling, vertical axis−EDV approximate). An approximate straight line is drawn through the origin. The formula for the approximate straight line and the coefficient of determination R^2^ are given in the figure. (**B**). Bland–Altman analysis (horizontal axis—average of EDV approximate and EDV coupling, vertical axis—difference between EDV approximate and EDV coupling, dashed line—limits of agreement (LOA)) with all data; a total of 42 values measured at 3 points each from 14 participants were used.

In terms of total points, a significantly high correlation was observed between EDV coupling and EDV approximate (R^2^ = 0.96, *p* < 0.05) (Figure 4A). The acceptable range of error was from −5.8 to 15 mL/m^2^. The percentage error for EDV coupling was 16.3% (Figure 4B).

## 4. Discussion

In this study, EDV was approximated using the mean blood pressure and SV, and the utility of EDV approximate was investigated by determining its correlation with the EDV coupling, calculated from Ees/Ea. The results show a very high correlation between EDV approximate and EDV coupling. In the past, EDV measured from Ees/Ea has been reported to correlate well with EDV (EDV echo) measured from transthoracic echocardiography (TTE) [6]. Therefore, it was suggested that EDV approximate had about the same accuracy as EDV echo. However, EDV approximate and EDV echo were not compared directly. Therefore we believe that it is acceptable to consider comparing EDV approximate and EDV echo to ensure accuracy.

In the present study, a normal value taken from the literature (2.3 mmHg/mL) [8,9] was chosen for Ees in relation to EDV in the approximation formula. As a result, both the EDV coupling value and the EDV approximate value were roughly in accord with the normal EDV values reported in the past, of 50 ± 12 mL/m^2^ (male) and 46 ± 9 mL/m^2^ (female), respectively [12]. These EDV values were measured in the healthy Japanese participants with TTE. Although the patient group was not exactly the same, the values were similar to those of EDV coupling or EDV approximate; hence, we could expect that the EDV coupling or EDV approximate would show a high correlation to EDV echo. In the present study, an Ees of 2.3 mmHg/mL [8,9] was selected, but whether a normal Ees value of 2.3 mmHg/mL is suitable or not needs to be investigated. In the present study, the mean value for EDV approximate was shown to be slightly higher than the value for EDV coupling. With the equation EDV approximate = Pm/Ees + SV, the Ees value we took could be smaller than the actual value; therefore, it may be possible that a larger value was calculated for EDV approximate. In the near future, we would like to investigate whether Ees should be obtained for each patient or not. It might be useful to investigate Ees before anesthesia induction. We can obtain Ees from the TTE when patients are awake. The equation for calculating Ees should be Ees = Pes/ESV. Using these Ees values (regarded as Ees awake for simplification) would reveal what value of Ees should be chosen for calculating a more accurate EDV approximate.

Sunagawa reported that Pes and Pm are about equal in terms of size [7], and in the present study as well, mean blood pressure was used instead of Pes. An arterial waveform is necessary to obtain Pes, but Pm can be measured from a manchette. In addition, applying Pm is also reasonable from the perspective of convenience and non-invasiveness. If left ventricular preload can be expressed using EDV approximate, invasive arterial pressure monitoring may no longer be necessary to ascertain left ventricular function.

When rapid infusion loading was performed in the present study, Pes increased due to the increase in EDV, and it was predicted that blood pressure would be elevated and heart rate would decrease. It was also believed that the cardiac contraction force and arterial afterload would be unchanged. In these patients, EDV was increased, and Pes was increased, but no significant changes were observed in blood pressure and heart rate. Arterial afterload decreased. This result could be due to the two contradictory changes of circulation dynamics. One is the dilation of the peripheral blood vessels that leads to a decrease in left ventricular afterload, while the other is the increase in left ventricular preload with the fluid trial. The dilation of the peripheral blood vessels could have happened due to the parasympathetic nervous system becoming dominant with anesthesia induction.

In the 14 patients investigated in the present study, a strong correlation was observed among the measured values, but the correlation coefficients differed depending on whether the regression line passed through the origin or not. Regardless of whether values were measured or approximated, they were all calculated on the assumption that V_0_ = 0. In addition, as V_0_ is believed to be nearly equal to zero in healthy hearts, compared with EDV or ESV, the fact that the regression line passed through zero was not inconsistent. However, the presence of a fixed error was suggested; therefore, further research is needed with regard to these lines passing through the origin.

The future outlook includes the possibility of approximating EDV only by manchette and transcutaneous oxygen saturation monitors. This can only be made feasible by using the SV of estimated continuous CO (esCCO) (Nihon Kohden, Tokyo, Japan). Arterial pressure monitoring will no longer be necessary. Written as V_0_ = 0 from EF = SV/EDV and EDV = Pes/Ees + SV + V_0_, this becomes Ees = Ea·EF/(1 − EF). From this equation, Ees can be obtained individually, and when EDV and Ea are obtained from mean blood pressure and esSV from esCCO monitor, not only arterial pressure waveform but also the phonocardiogram will become unnecessary. Additionally, left ventricular function can be assessed less and less invasively. Arterial blood pressure, CV catheter, or Swan–Gantz catheter will not be needed for understanding left ventricular function.

This study has several limitations. First, this was a prospective study, but investigations were conducted at a single institution. We may consider investigating with several institutions for achieving more accurate results. As conducted in a single institution, variations between patients could not be completely eliminated, and the number of participants was insufficient to adjust for conditions, such as physique, age, and whether the patients had epidural anesthesia. We are now undertaking other research on fluid challenge, and expecting that we will be able to adjust patients’ conditions next time. Second, we excluded cases in which vasoconstrictors were administered during an approximate 1 h period, including the 15 min before and after the infusion load, but we could not completely rule out the influence of changes in Ees and Ea in cases in which vasoconstrictors were administered earlier. For example, if a vasopressor, such as ephedrine, is administered 20 min before fluid challenge, we cannot ensure that there is no effect on circulation dynamics at the point of the fluid challenge. We cannot determine that the increase in the EDV is caused only by fluid infusion, as there will be a suspicion that the changes may have been caused by ephedrine. Therefore, to fully eliminate the effect of vasopressors, we should perform the fluid challenge without administering vasopressors even if the blood pressure becomes lower than the tolerable limit. However, this cannot happen because trials are performed in human beings. Third, we cannot completely isolate the circulation dynamics. In an actual heart, hemodynamic changes occur in relation to fluctuations in Ees and Ea, and a new end systole point is produced. SV changes according to this, and EDV is also believed to increase. There is a complex interaction among left ventricular preload, contraction, and afterload. Regarding Ees as a fixed value, it may be too rough to decipher circulation dynamics. Using a fixed Ees value of 2.3 mmHg/mL could lead to the oversimplification of EDV. Fourth, because EDV was calculated based on the SV obtained from the FloTrac sensor, it could not be measured in the time frames when extrasystole occurred, and the analysis was not possible. This limitation may be solved using esCCO; however, it is necessary to investigate the accuracy of esSV in patients with extrasystole first, such as arterial fibrillation, premature arterial contraction, or premature ventricular complex. Fifth, using this method, V_0_ was assumed to be zero. V_0_ was taken to be zero because the participants were patients with normal heart function [13,14]. Patients with poor heart function may not have a negligible amount of V_0_ because of the dysfunction of their hearts.

We are considering clinical practice using EDV approximate. This method allows for the easy monitoring of left ventricular EDV using MAP and SV. The EDV approximate value transition can be displayed in the anesthesia record to evaluate changes during general anesthesia and to understand fluctuations in circulating blood volume. The EDV approximate value transition allows us to control the infusion rate to maintain the left ventricular preload. However, it is unclear whether the goal should be to bring the approximate value of EDV close to the universal normal value, or to seek a control value after the induction of general anesthesia. We would like to reexamine this issue in the future.

## 5. Conclusions

The findings of this study suggest that EDV approximated from mean blood pressure and SV with a fixed value of Ees shows high correlation between EDV calculated from Ees/Ea, and also suggest that the non-invasive and continuous monitoring of EDV can be performed from mean blood pressure and SV during general anesthesia.

## Figures and Tables

**Table 1:** Patients’ characteristics.

Characteristics	Value
Age, years	63 ± 14
Gender, male/female, %	50%/50%
Height, cm	162 ± 8
Weight, kg	62 ± 11
BMI, kg·m^-2^	24 ± 8
SBP, mmHg	95 ± 16
DBP, mmHg	48 ± 9
PEP, second	86 ± 62
ET, second	330 ± 60
EDV coupling, mL·m^−2^	51 ± 11
EDV approximate, mL·m^−2^	56 ± 7
SV, mL	64 ± 10

## Data Availability

The data that support the findings of this study are available from the corresponding author, upon reasonable request.
